# Supplementary material for: IgA2 Antibodies against SARS-CoV-2 Correlate with NET Formation and Fatal Outcome in Severely Diseased COVID-19 Patients
Source: Cells. 2020 Dec 12;9(12):2676. doi: 10.3390/cells9122676 (PMC7764693; doi:10.3390/cells9122676)
Supplement: Supplementary file 1 [file cells-09-02676-s001.pdf]

cells

MDPI

Communication

Supplementary Materials: IgA2 Antibodies against SARS-CoV-2 Correlate with NET Formation and Fatal Outcome in Severely Diseased COVID-19 Patients

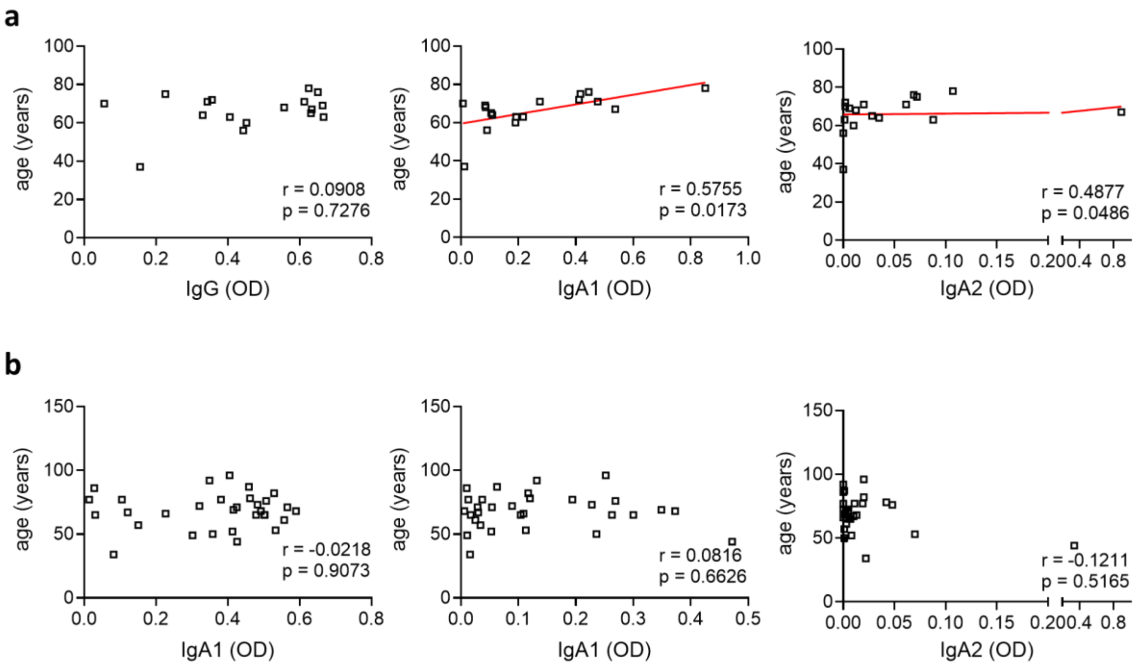

Figure S1. SARS-CoV-2 specific IgA1 and IgA2 levels correlate with age in severely diseased patients. Correlation of levels of IgG, IgA1 and IgA2 directed against SARS-CoV-2 with age in the plasma of SARS-CoV-2-infected patients with severe (**a**) (N = 17) or moderate (**b**) disease (N = 31). Significances were tested with with Spearman's correlation coefficient. *p < 0.05; **p < 0.01; and ***p < 0.001. Data are presented as scatter plots with mean ± s.e.m. or scatter plots.

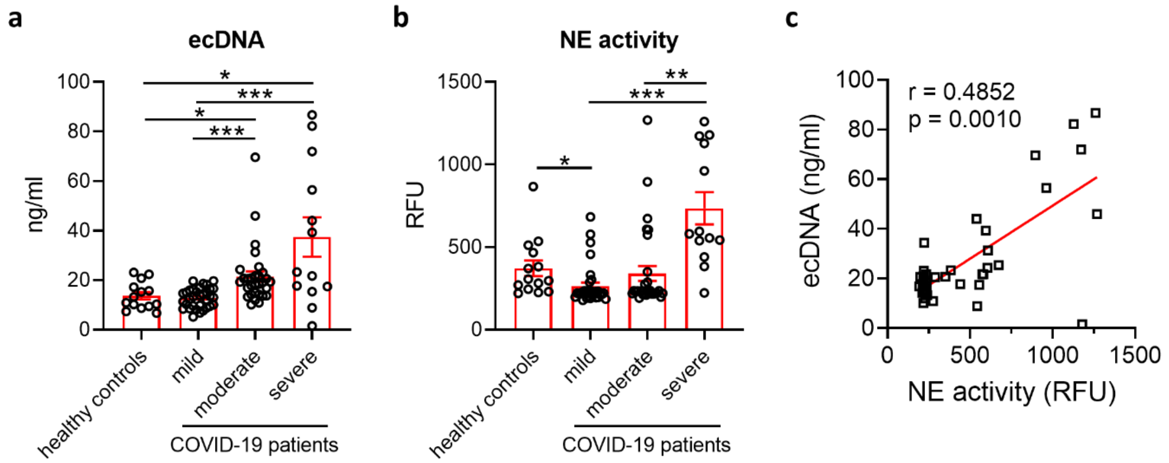

Figure S2. ecDNA and NE activity are elevated in patients with moderate and severe disease. (**a**,**b**) Amounts of (**a**) ecDNA and (**b**) NE activity in the plasma of healthy control subjects (N = 14) as well as SARS-CoV-2-infected patients with no or mild disease symptoms (N = 33) moderate symptoms (N = 30) or severe symptoms requiring intensive care treatment (N = 13). (**c**) Correlation of scDNA with NE activity in the plasma of SARS-CoV-2-infected patients with moderate or severe symptoms (N = 43). Significances were tested with Kruskal-Wallis test followed by Dunn's multiple comparison test for all groups (**a**+**b**) and with Spearman's correlation coefficient (**c**). *p < 0.05; **p < 0.01; and ***p < 0.001. Data are presented as scatter plots with mean ± s.e.m. or scatter plots.
